# Childbirth without Pain

**Published:** 1879-03-20

**Authors:** R. B. Anderson

**Affiliations:** Georgia


### *CHILDBIRTH WITHOUT PAIN.*

BY R. B. ANDERSON, M.D., OF GEORGIA.

Two remarkable cases of painless labor, within the last twelve years, have occurred in my practice; the first in March, 1866. Mrs. ——, of Milton county, Ga., expecting to be confined and having passed through her pregnancy up to time, as usual, it being the sixth pregnancy without any unfavorable symptoms, and no signs of labor, I thought I could be absent for one night from the neighborhood. Consulted her as to the propriety of leaving, and engaged an old lady who had followed the business for many years, living just across the road from her, to watch over her and if labor should come on in my absence, and there should be the least bad symptom, to call in my friend, W. P. P., immediately.

On the second day, about sunset, she experienced a bearing-down sensation, and thought best to call the old lady over to stay with her until my arrival. The bearing sensation increased, having regular intervals, for about two hours, at which time she got in bed, and the first bearing-down sensation (which was almost instantly), the child was delivered, before anyone could get to her to assist her in the least; in a few minutes the placenta was thrown off in the same way, without the least pain. The uterus contracted finely, there was no hemorrhage or after pains during that night, though after that night she experienced after-pains as usual. I arrived at the place about one hour after the birth of the child.

The next case was that of a colored woman who had aborted several times, within the last ten years; had never carried a child to full term, nor longer than from two to five months. I was called to see her on the 31st of January last; at one o'clock A.M.; found her in labor, pains bearing down; the child presented; the os dilated; the waters had escaped. She informed me that she had been suffering pain for three weeks, and that for more than twelve hours her pains had been severe, and that they had been bearing down for two or more hours. I watched the case, its progress, etc., for one hour, when she remarked that the child had gone back; this she did just as a pain was going off. I then made examination and found the child's head had receded, at least 2½ or 3 inches. At this time she complained of severe pain above the symphysis pubis; her complaint rather led me to fear laceration of the uterus anteriorly, as the head of the child was then nearly in reach, whereas, before it was almost pressing the perineum. I examined the abdomen to see if I could detect, through the abdominal parietes, any portion of the child that might have escaped into the abdominal cavity. I also examined the pulse; all appeared right; no evidence in either showing the least sign of laceration. I waited 2 or 3 pains without the least advancement of the child. I then placed her on the lap, and a pain or two brought the child to its former position. She soon became faint and begged to be put to bed. This being done, her pains continued as before, without the slightest advancement. After waiting for sometime, I had her again placed on the lap; her pains ceased entirely; contraction of the uterus continued to recur as when she had pains, but without the slightest pain. Placing my hand upon the abdomen I could feel the contraction, and would ask her at every contraction if she felt any pain; her answer was invariably: "No, sir, not the slightest pain, I only feel a drawing and pressing down, but not painful." Thus she went on until delivered of a large, healthy-looking male child; the placenta came also without pain, neither had she any after-pains, and she is now, February 8, doing well.

On the 4th inst., a medical friend of mine informed me that he delivered a colored woman of her 14th child, who declared to him that she had never suffered pain in childbirth. The colored woman, mother of the 14 children, is now living in Marietta, where she gave birth to her last child.

